# Supplementary material for: Cross-informant ratings on emotional and behavioral problems in Nepali adolescents: A comparison of adolescents’ self-reports with parents’ and teachers’ reports
Source: PLoS One. 2024 May 16;19(5):e0303673. doi: 10.1371/journal.pone.0303673 (PMC11098339; doi:10.1371/journal.pone.0303673)
Supplement: Attachment — Submitted filename: Response to Reviewer.docx [file pone.0303673.s002.docx]

**Response to the Reviewers’ comments**

Dear Reviewers,

We would like to thank you for reviewing our manuscript. Your comments and questions have been valuable in improving the quality of our paper. We have revised the manuscript according to your advice, to the best of our knowledge. References have been revised accordingly. We have also done additional proofreading, correcting some words and sentences, but without changing the meanings and not adding any more information to the text.

Below, we have given our answers to your comments one by one. In doing so, we have referred to the corresponding lines in our revised manuscript with track changes (see *Revised manuscript with track changes*).

**Reviewer #1**

**Reviewer’s comment**

1. In the abstract, results, and conclusion, similarities can be acknowledged. The author may include recommendations in the conclusion.

**Our Response:**

Thank you for the suggestion. We have now revised the conclusion part of the abstract, including implications for clinical practice – see lines 46-52.

**Reviewer’s comment**

2. The introduction section of the full paper lacks a statement of the problem for the current study.

**Our Response:**

Thank you for the comment. We have now included the statement of problems for the current study, see the last paragraph of the introduction, lines 131 – 132, 138-145.

**Reviewer’s comment**

3. In line 136, the third aim employs the word "exploring," which appears to be more qualitative. This word should be reframed.

**Our Response**

Thank you for the suggestion. We agree and have now changed the word “exploring” to “examining”, see line 149.

**Reviewer’s comment**

4. Cronbach's alpha should be mentioned in the Youth Self Report, Child Behavior Checklist, and Teacher’s Report Form.

**Our Response:**

Thank you for the suggestion. We have added Cronbach’s alpha for the syndrome scales of the Child Behavior Checklist/6-18 years, and Teacher’s Report Form/6-18 years, in lines 217-221. In addition, for the reader's convenience and to provide a comprehensive overview of the psychometric properties of the syndrome scales across different measures, we have included a compiled table of Cronbach's alpha values, see lines 230-235.

**Reviewer’s comment**

5. Please use a more appropriate term in line 481; instead of "mental symptoms," it should be reframed.

**Our Response:**

Thank you for the comment. We have now used “mental health problems” instead of “mental symptoms” in the corresponding line (see line 597).

**Reviewer’s comment**

6. Strengths and recommendations should be emphasized more in terms of further research scope in the conclusion section.

**Our Response:**

Thank you for the comment. We have rearranged and extended the limitation part in the “ Strengths and Limitations” paragraph, see lines 606-663. Further, we have moved the recommendations for further research from the Strengths and Limitations part to a new paragraph called “Future research” to emphasize our recommendations, see lines 660-667.

**Reviewer’s comment**

7. Negative results can also be included in the discussion section.

**Our Response:**

Thank you for the comment. We have now included negative findings in the discussion section in lines 530-532 “*It should be mentioned that in the present study, we did not find any impact other factors, such as the age of the adolescents and the experience of negative/traumatic life events on cross-informant discrepancies”*, and also in lines 588-589 as “*However, the teacher-adolescent discrepancies were not associated with adolescents’ experience of family stress/conflicts.”*

**Reviewer #2**

**Reviewer’s comment**

The manuscript delves into a pertinent theme and is well articulated. However, it falls short of being entirely groundbreaking, especially given its focus on a specific population of Nepalese adolescents. This aspect wasn't adequately addressed in the data discussion. Moreover, the clinical implications of the observed low agreement among adolescents, their parents, and teachers concerning emotional and behavioral problems require further exploration.

**Our Response:**

Thank you for your comments. In the Strengths and Limitations part of the Discussion section, we have argued that the study is the first large-scale study of its kind in Nepal, that it was conducted in many districts in different parts of the country, and that the participation rate was exceptionally high, in lines 600-605. However, we have commented more as to the limitations of the study sample, see lines 606-633. Further, we have commented more on the clinical implications of the low cross-informant agreement in the Clinical Implications part of the Discussion section, see lines 634-659.

**Reviewer’s comment**

The statistical analyses conducted are appropriate and align with the study's objectives, albeit being relatively straightforward (t-test, correlation, and regression). I suggest including descriptive analyses of emotional and behavioral problem indicators to comprehend the percentage of the sample classified as normal, borderline, or clinical. Additionally, a comparison of assessments between parents and teachers regarding adolescents' emotional and behavioral problems is recommended. Teachers, with their close interaction and potential to observe changes, are crucial in mental health referrals.

**Our Response:**

Thank you for the comments. We agree that examining the prevalences of adolescent EBPs by presenting data from the YSR, CBCL, and TRF might be of interest. However, we chose to compare the mean scores (magnitude of problems) only, as Nepali norms do not yet exist, and the estimates would be based on available American norms. As the validity of our findings when using the American norms might be questionable as to relevant cut-off points, we decided to use the raw scores (mean scores) that are independent of cultural norms. Hopefully, future studies may provide appropriate norms for Nepali children and adolescents.

Another thing is that if we extend the study to include prevalence tables and comments/discussion about those findings as well, it might make the study less focused on its main topic, i.e., the cross-informant correlations. We would like to inform you that in our preceding paper on self-reported EBPs among school-going adolescents in Nepal published in 2023 (same sample), we already presented adolescent-reported prevalences and percentages classified as normal, borderline, and clinical, and for all problem scales (Adhikari S, Ma J, Shakya S, Brøndbo PH, Handegård BH, Javo AC. Self-reported emotional and behavioral problems among school-going adolescents in Nepal - A cross-sectional study. PLOS ONE. 2023;18(6):e0287305. doi: 10.1371/journal.pone.0287305. PMID: 37352299). The reason for not including a parent-teacher comparison of adolescent EBPs is that we wanted to avoid replication. A previous paper by Ma and colleagues had already examined parents’ and teacher’s reports for the wider age group of children and adolescents (6-18 years), which also included the present study sample of adolescents (Ma, J., Mahat, P., Brøndbo, P.H. et al.: *Teacher reports of emotional and behavioral problems in Nepali schoolchildren: to what extent do they agree with parent reports?* BMC Psychiatry 22, 584 (2022). https://doi.org/10.1186/s12888-022-04215-4).

**Reviewer’s comment**

Lastly, it's noteworthy that the question about serious life events/trauma used in the regression analysis was formulated by the authors and is not part of a validated questionnaire, potentially weakening the analysis.

**Our Response:**

Thank you for rightly pointing out that limitation. We now mentioned that in the limitations section, lines 623-627.

**Reviewer’s comment**

The manuscript is written in clear and cohesive language. However, I recommend organizing the text more integratively, emphasizing the interrelation among the various aspects considered in the study. This would facilitate a more comprehensive discussion.

**Our Response:**

Thank you for your constructive feedback. We have now reorganized the Discussion section to improve text integration. The first paragraph now includes the comparison of mean scores of parents, teachers, and the adolescents themselves. The next paragraph focuses on correlations. Instead of two separate discussions under separate headings, one about the parent-adolescent agreement and one about the teacher-adolescent agreement, we have now merged the discussions under one common heading (see lines 369-446).

**Reviewer’s comment**

Out of the 65 references cited, only 23 have been published in the last five years. Furthermore, several references lack complete data, such as DOIs. Providing this information is crucial to facilitating readers' access to the cited documents. I recommend ensuring that all references, including DOIs, are complete to enhance the comprehensiveness and accessibility of the bibliography.

**Our Response:**

Thank you for the comment. We have now completed the bibliography section with all required data, such as DOI, wherever applicable. We also have added a few new references in the text (lines 67, 104, 404, 439, 612) and the references (lines 719-726, 785-789, 856-859, 868-870, 920-922.
