# Supplementary material for: Cross-informant ratings on emotional and behavioral problems in Nepali adolescents: A comparison of adolescents’ self-reports with parents’ and teachers’ reports
Source: PLoS One. 2024 May 16;19(5):e0303673. doi: 10.1371/journal.pone.0303673 (PMC11098339; doi:10.1371/journal.pone.0303673)
Supplement: Attachment — Submitted filename: COMMENTS.docx [file pone.0303673.s003.docx]

| Overall, it is a well-conducted study. |  |  |
| --- | --- | --- |
| Cross-informant correlations on emotional and behavioral problems in Nepali adolescents. Do problems reported by adolescents differ from those reported by their parents and teachers? | The title requires modification. |  |
| Based on the population distribution of  the three main ecological/geographical regions of Nepal, sixteen districts were selected (three from  the Mountains region, six from the Middle Hills region, six from the Terai region, and the  Kathmandu district) | Any sampling procedure used for the selection of district | In the methodology section, the sampling procedure appears to be weak; utilizing multistage sampling or probability proportionate to size sampling would have been more robust |
| Six adolescents  (three boys and three girls aged 11-18 years) from each grade level (grades 6-10) were randomly  selected using random number tables, regardless of caste/ethnicity or other background variables.  The overall participation rate was 99.2% for the adolescent sample and parent sample, and 99.1%  for the teacher sample. | What scientific rationale supports the selection of only six students from each grade? |  |
|  |  |  |
| Statistical analysis | The researcher opted for Pearson's correlation, but employing the intraclass correlation coefficient (ICC) would have been more advantageous. | COMMENTS  Using the intraclass correlation coefficient (ICC) instead of Pearson's correlation coefficient can indeed be more appropriate in certain research contexts, particularly when dealing with data that involve multiple raters or measurements on the same subjects.  Pearson's correlation coefficient measures the strength and direction of the linear relationship between two variables. It assumes independence of observations and is primarily used for assessing the relationship between two continuous variables.  On the other hand, the ICC is a statistical measure used to assess the reliability and consistency of measurements made by multiple observers or raters, or by the same observer or rater on different occasions. It is particularly useful in situations where there is a need to quantify the agreement or reliability among different raters or between different measurements made on the same subjects.  In research settings where there are multiple raters providing ratings or measurements on the same set of subjects, ICC provides a more robust measure of reliability compared to Pearson's correlation coefficient. It takes into account both systematic and random variations in the data and provides a measure of consistency or agreement among the raters or measurements.  Therefore, if the researcher is dealing with data that involve multiple raters or repeated measurements, using ICC would likely provide a more appropriate measure of reliability compared to Pearson's correlation coefficient. However, the choice between these two measures ultimately depends on the specific research question and the nature of the data being analyzed. |
