# Supplementary material for: Cross-informant ratings on emotional and behavioral problems in Nepali adolescents: A comparison of adolescents’ self-reports with parents’ and teachers’ reports
Source: PLoS One. 2024 May 16;19(5):e0303673. doi: 10.1371/journal.pone.0303673 (PMC11098339; doi:10.1371/journal.pone.0303673)
Supplement: Attachment — Submitted filename: Response to Reviewer.docx [file pone.0303673.s004.docx]

**Response to Reviewer**

| **Reviewer’s comment** | Overall, it is a well-conducted study. |  |  |
| --- | --- | --- | --- |
| **Our response** | Thank you for the positive comment.  . | | |
| **Reviewer’s comment** | Cross-informant correlations on emotional and behavioral problems in Nepali adolescents. Do problems reported by adolescents differ from those reported by their parents and teachers? | The title requires modification. |  |
| **Our response** | Thank you for the comment. We have changed the title to a more appropriate one now.: *“**Cross-informant ratings on emotional and behavioral problems in Nepali adolescents: comparison of adolescents’ self-reports with parents’ and teachers’ reports”*. See lines 1-3 in the manuscript. | | |
| **Reviewer’s comment** | Based on the population distribution of  the three main ecological/geographical regions of Nepal, sixteen districts were selected (three from  the Mountains region, six from the Middle Hills region, six from the Terai region, and the  Kathmandu district) | Any sampling procedure used for the selection of district. | In the methodology section, the sampling procedure appears to be weak; utilizing multistage sampling or probability proportionate to size sampling would have been more robust. |
| **Our response** | Thank you for pointing out possible weaknesses of the sampling procedure used to select districts. We selected districts as we selected schools using a purposive sampling method based on accessibility and feasibility. We have now clarified it in the manuscript and explained the reasons for choosing this method in lines 157-160. We acknowledge that the random selection of districts and schools using probability sampling would have been more robust. However, this was a large, countrywide study that required an extensive amount of time and money to accomplish. Therefore, we chose the purposive sampling technique for cost-effectiveness and ease of data collection and travel. Nevertheless, we randomly selected the students from each grade using simple random sampling. As we recognize that purposive sampling to select districts and schools was a limitation of our study, we have also added a few more lines about it in the limitation section, lines 520-522. | | |
| **Reviewer’s comment** | Six adolescents  (three boys and three girls aged 11-18 years) from each grade level (grades 6-10) were randomly  selected using random number tables, regardless of caste/ethnicity or other background variables.  The overall participation rate was 99.2% for the adolescent sample and parent sample, and 99.1%  for the teacher sample. | What scientific rationale supports the selection of only six students from each grade? |  |
| **Our response** | We selected six students only from each grade for a couple of reasons. One reason was that some government schools in Nepal, mostly in rural areas, have very few students (less than 10 students per class), whereas private schools usually have more students per class. To ensure an equal number of students from both government and private schools, we decided on a smaller number of students from each grade. Another reason was to keep the burden of filling out the TRF reasonable for teachers, who would have to answer more than 150 questions per student. An additional reason to keep the number of students per classroom low was our concern about the dependency of scores from clustering of students within classrooms when the same teacher informs about multiple students. Sampling many students within a classroom increases the design effect, and thus reduces the potential benefit of increased within-classroom sample size. | | |
| **Reviewer’s comment** | Statistical analysis | The researcher opted for Pearson's correlation but employing the intraclass correlation coefficient (ICC) would have been more advantageous. | COMMENTS  Using the intraclass correlation coefficient (ICC) instead of Pearson's correlation coefficient can indeed be more appropriate in certain research contexts, particularly when dealing with data that involve multiple raters or measurements on the same subjects.  Pearson's correlation coefficient measures the strength and direction of the linear relationship between two variables. It assumes independence of observations and is primarily used for assessing the relationship between two continuous variables.  On the other hand, the ICC is a statistical measure used to assess the reliability and consistency of measurements made by multiple observers or raters, or by the same observer or rater on different occasions. It is particularly useful in situations where there is a need to quantify the agreement or reliability among different raters or between different measurements made on the same subjects.  In research settings where there are multiple raters providing ratings or measurements on the same set of subjects, ICC provides a more robust measure of reliability compared to Pearson's correlation coefficient. It takes into account both systematic and random variations in the data and provides a measure of consistency or agreement among the raters or measurements.  Therefore, if the researcher is dealing with data that involve multiple raters or repeated measurements, using ICC would likely provide a more appropriate measure of reliability compared to Pearson's correlation coefficient. However, the choice between these two measures ultimately depends on the specific research question and the nature of the data being analyzed. |
| **Our response** | Thank you for the comment. We acknowledge that using the intraclass correlation coefficient (ICC) instead of Pearson's correlation coefficient would have been more advantageous if the focus of the study was on assessing the reliability of the measurements. In a situation where the raters view the adolescent from the same angle (for example in an observational situation or from a video recording) using the same set of items, it would be more natural to report reliability. However, in the specific context of our study which primarily focused on examining the agreement among different informants, such as teachers, parents, and adolescents, who view the child’s behavior in different settings and from different “angles”, we believe that reporting Pearson’s correlation remains the more appropriate choice. Furthermore, the study employed three different measures, namely the Youth Self-Report (YSR), Child Behavior Checklist (CBCL), and Teacher's Report Form (TRF), each with distinct sets of items tailored to capture adolescents' emotional and behavioral problems from different perspectives. Although ICC does not explicitly assume homogeneity of items within different measures, the interpretation can be influenced when the items are markedly different across measures, such as in the YSR, CBCL, and TRF. Therefore, we chose an alternative measure of agreement, such as Pearson's correlation coefficient, which we believe is more appropriate for our study. | | |
